# Evaluating the effectiveness and cost effectiveness of the ‘strengthening families, strengthening communities’ group-based parenting programme: study protocol and initial insights

**DOI:** 10.1186/s12889-021-11912-4

**Published:** 2021-10-19

**Authors:** Annemarie Lodder, Anita Mehay, Hana Pavlickova, Zoe Hoare, Leandra Box, Jabeer Butt, Tim Weaver, Mike J. Crawford, Donna Clutterbuck, Nicola Westbrook, Karlet Manning, Saffron Karlsen, Steve Morris, Andrew Brand, Paul Ramchandani, Yvonne Kelly, Anja Heilmann, Richard G. Watt

**Affiliations:** 1 Department of Epidemiology and Public Health, University College London, London, UK; 2 NWORTH Clinical Trials Unit, Bangor University, Bangor, UK; 3 Race Equality Foundation, London, UK; 4 School of Health and Education, Middlesex University, London, UK; 5 Division of Psychiatry, Imperial College London, London, UK; 6 PPI lead, Manchester, UK; 7 Centre for the Study of Ethnicity and Citizenship, University of Bristol, Bristol, UK; 8 Department of Public Health & Primary Care, Cambridge University, Cambridge, UK; 9 PEDAL Research Centre, Faculty of Education, Cambridge University, Cambridge, UK

**Keywords:** ‘Parenting’, ‘Child outcomes’, ‘Parental well-being’, ‘Health inequalities’, ‘Randomised controlled trial’, ‘Parenting programme’, ‘Child well-being’, ‘Intervention’

## Abstract

**Background:**

Up to 20% of UK children experience socio-emotional difficulties which can have serious implications for themselves, their families and society. Stark socioeconomic and ethnic inequalities in children’s well-being exist. Supporting parents to develop effective parenting skills is an important preventive strategy in reducing inequalities. Parenting interventions have been developed, which aim to reduce the severity and impact of these difficulties. However, most parenting interventions in the UK focus on early childhood (0–10 years) and often fail to engage families from ethnic minority groups and those living in poverty. *Strengthening Families, Strengthening Communities (SFSC)* is a parenting programme designed by the Race Equality Foundation, which aims to address this gap. Evidence from preliminary studies is encouraging, but no randomised controlled trials have been undertaken so far.

**Methods/design:**

The TOGETHER study is a multi-centre, waiting list controlled, randomised trial, which aims to test the effectiveness of SFSC in families with children aged 3–18 across seven urban areas in England with ethnically and socially diverse populations. The primary outcome is parental mental well-being (assessed by the Warwick-Edinburgh Mental Well-Being Scale). Secondary outcomes include child socio-emotional well-being, parenting practices, family relationships, self-efficacy, quality of life, and community engagement. Outcomes are assessed at baseline, post intervention, three- and six-months post intervention. Cost effectiveness will be estimated using a cost-utility analysis and cost-consequences analysis. The study is conducted in two stages. Stage 1 comprised a 6-month internal pilot to determine the feasibility of the trial. A set of progression criteria were developed to determine whether the stage 2 main trial should proceed. An embedded process evaluation will assess the fidelity and acceptability of the intervention.

**Discussion:**

In this paper we provide details of the study protocol for this trial. We also describe challenges to implementing the protocol and how these were addressed. Once completed, if beneficial effects on both parental and child outcomes are found, the impact, both immediate and longer term, are potentially significant. As the intervention focuses on supporting families living in poverty and those from minority ethnic communities, the intervention should also ultimately have a beneficial impact on reducing health inequalities.

**Trial registration:**

Prospectively registered Randomised Controlled Trial ISRCTN15194500.

## Background

Up to 20% of children and adolescents in the United Kingdom experience socio-emotional difficulties [1] which have serious implications for the individuals affected, their families and wider society. The health and well-being of children and adolescents is influenced by a complex array of inter-related factors. Parents have a fundamental influence on their child’s development, health and well-being, and parental mental health has a profound effect on family life, relationships and parenting practices [2]. Data from the UK Millennium Cohort Study show that approximately a third of mothers (33%) and fathers (30%) reported depressive symptoms [3]. Stark socio-economic and ethnic inequalities exist for both socio-emotional difficulties in childhood and mental health problems in parents [4–8]. Notably, poverty can contribute to parental stress, depression and irritability leading to disrupted parenting and to poorer outcomes for children [9]. Consequently, giving every child the best start in life has been identified as a key public health strategy to combat health inequalities [5].

An important element of this strategy is supporting parents to develop effective parenting practices and skills. Parenting programmes are effective in improving parenting skills and mental health [10, 11], which appear to mediate improvements in child behaviour problems [12]. Several Cochrane reviews of universal and targeted group-based parenting programmes have demonstrated a variety of positive effects on both child and parental outcomes [10, 13–15]. Parenting programmes appear to be effective for parents regardless of trial setting and severity of problems at baseline, suggesting that a range of families can engage and benefit from these programmes [10, 16].

There is a global interest in evidence-based parenting programmes [12] with increasing attention in the UK where policy-makers seek proven and cost-effective methods of improving child well-being. Existing Cochrane reviews have highlighted the need for larger scale and well-designed trials that target older children and families from ethnic minority groups as well as socially disadvantaged backgrounds, and also include comprehensive economic evaluations [17]. *Strengthening Families, Strengthening Communities* (SFSC) is one such group-based parenting intervention designed to support parents with children up to 18 years old. SFSC has been particularly used to support families from poorly served marginalised communities, including ethnic minority parents, teenaged parents and fathers [18]. SFSC has been delivered by community organisations and a range of statutory organisations across the country since 2000 with the support of the Race Equality Foundation.

Several uncontrolled studies have evaluated the programme and demonstrated encouraging positive outcomes for both children and parents [19–22]. However, to date no randomised controlled trials (RCTs) have been conducted. A trial evaluating SFSC will directly address the knowledge gaps identified in the Cochrane reviews of parenting interventions [10, 13–15] and will focus on families with older children, specifically targeting families from ethnically and socially diverse communities. The TOGETHER study aims to assess the effectiveness and cost effectiveness of the SFSC programme in enhancing parental mental well-being and children’s social and emotional well-being up to 6-months post intervention across urban areas in England. There are two stages of this study: an internal pilot (already completed) and the main trial. We have now entered the main trial and this paper aims to describe the study protocol of the randomised controlled trial but will also describe the initial challenges undertaking the study, particularly during the COVID-19 pandemic, and how these were addressed.

## Methods/design

### Design

This study is a multi-centre, waiting list controlled, randomised trial designed to assess the effectiveness and cost effectiveness of SFSC programmes. The study is conducted in two stages: Stage 1 comprised a 6-month internal pilot to determine the feasibility of the trial. The study is currently in Stage 2: recruiting for the main trial.

An embedded mixed methods process evaluation will assess the intervention fidelity, support the internal pilot by assessing the acceptability of trial procedures, and investigate the experience of those delivering and receiving the intervention during the full trial, yielding insights into the influence of contextual factors on SFSC delivery and outcome generation. The Recommendations for Interventional Trials (SPIRIT) checklist was used to structure the study’s protocol.

### Settings

The study takes place in community settings across seven urban areas in England where the SFSC programme has successfully engaged with parents from disadvantaged socio-economic backgrounds, as well as a range of ethnic backgrounds. Working in close partnership with the Race Equality Foundation, these areas have been selected as they are the locations where the SFSC intervention is currently commissioned and delivered. The delivery agents within these areas are usually Local Authorities or community organisations who have been commissioned by Local Authorities to deliver SFSC within a specific locality.

### Participants

The SFSC programme is designed to reach a wide range of parents with children aged up to 18 years, including both mothers and fathers, lone parents, families living in deprived neighbourhoods and parents from minority ethnic communities. Parents attending the programme include self-referrals and those referred by social work, health, family support or criminal justice professionals. Data from 2014 to 2017 [4, 23] collected by the Race Equality Foundation provide a profile of the diversity of participants attending SFSC programmes, where over 40% were lone parents, around half were from minority ethnic communities and for nearly 70%, secondary school was their highest educational level. Recognising that an important feature of the SFSC programme is its universal and inclusive nature, the inclusion and exclusion criteria are kept as non-restrictive as possible.

#### Inclusion criteria

- Parents or any person with parenting responsibilities for the index child aged 3–18 years, including biological parents, step parents, foster parents and legal guardians (herein referred to as ‘parent’).

#### Exclusion criteria

- Unwilling to provide written informed consent to participate.
- Already participating in another interventional research study.
- Parents where there is an active court proceeding relating to separation between parent and child.

### Randomisation

Randomisation is via a secure online system using a sequentially randomised dynamic adaptive algorithm [24], stratified by site, gender of parent, and self-referral status, with a 1:1 allocation ratio. Participants are randomised by a member of the research team after consent and baseline questionnaires have been completed. To maintain blinding, the researcher randomising the participant will not be involved in collecting follow-up data. All researchers involved in follow-up data collection will be asked to complete a questionnaire to determine their perception of group allocation for each participant.

### Recruitment

Research sites recruited into the trial will be asked to designate specific SFSC programmes for the study.

#### Recruitment and consent process

##### Step 1: referral into the service

Participants either self-refer or will be recruited via existing referral pathways into the service either through current service waitlists or current referrals from other agencies such as social work, family support or criminal justice professionals. Parents who self-refer have either responded to promotional advertisements of the programme or have referred themselves after attending an outreach event (e.g., a coffee morning at a school). For the SFSC programmes involved in the study, programme staff will initially give general information about the study, discuss what the study involves and check the parent’s eligibility. If parents are interested, staff will gain verbal consent for their contact details to be passed on to the research team. A researcher will then contact the parent via their preferred method (phone, text, email) to assess whether they are interested and eligible to take part in the study and if so, to arrange either a face to face or online appointment to conduct the baseline interview.

##### Step 2 – baseline interview

During the initial meeting, the researcher will confirm eligibility, provide the participant information sheet either electronically or in paper format, and obtain informed consent (written or digitally recorded). Once consent has been gained, the baseline questionnaire interview will be conducted and on completion, the participant will be randomised. The parent will be told of their allocation immediately, and will receive written confirmation of their allocation in the form of a letter. Parents who are not eligible, or decline participation are referred back to the service. All participants will receive a debriefing leaflet outlining a list of national and local services on offer such as mental health charities, family support services and national helpline numbers.

##### Step 3 – commencement on SFSC programme

**Fig. 1:**
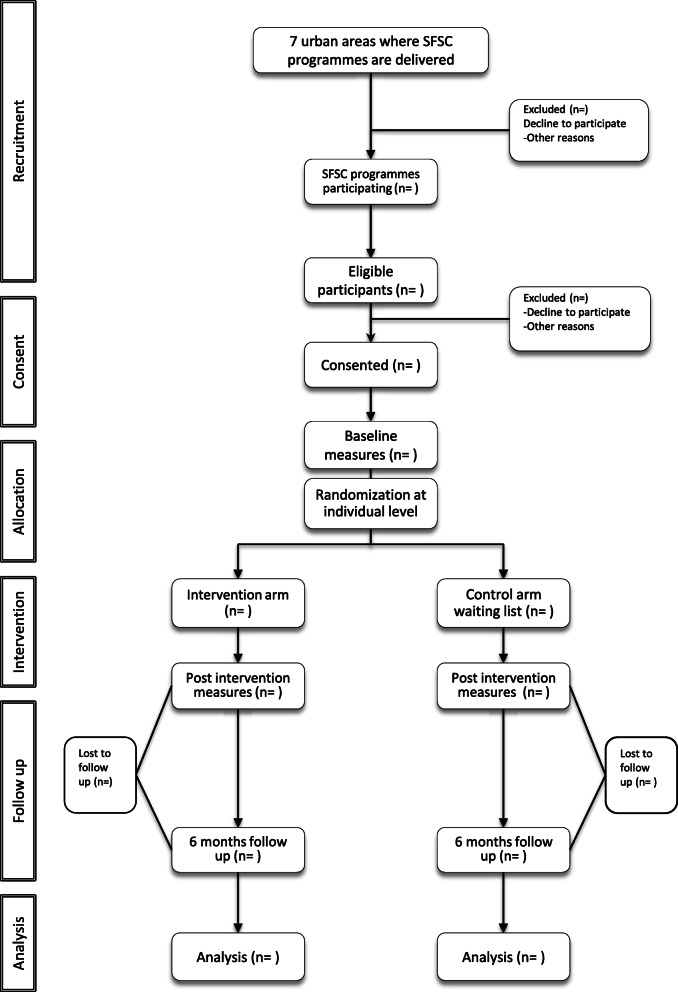
Study Flow Chart

Participants randomised to the intervention arm will enrol on the next programme available. Recruitment usually starts 8 weeks before the start of the programme. Those randomised to the control arm will be placed on a 10-month wait list. The study flow chart is outlined in Fig. 1*.*

#### Language support and translation

In areas with a high proportion of families from ethnic minority groups, Local Authorities and community organisations often provide SFSC groups delivered in a language other than English by trained SFSC facilitators. Based on the Race Equality Foundation experience, we expect that 50% of parents attending the SFSC programmes in the trial will be from an ethnic minority group. Of these, about 10% will require language support as they will not be able to complete the programme or research interviews in English. The most common languages at the research sites are expected to be Turkish, Somali, Sylheti, Bengali and Arabic.

To enable people who require language support to take part in the study, we will provide translated versions of research interviews and all research documents that are given to potential participants (such as, consent form, participant information sheet, letters confirming participation, advert leaflets). We will employ and train researchers fluent in the relevant languages to carry out the research interviews.

To translate research interviews, we have developed a robust translation process in line with World Health Organisation guidance [25] to achieve cross-cultural and conceptual, rather than merely linguistic, equivalence. This process will consist of:

- Step 1 – Forward Translation:
  - The source measure is forward translated by a professional translation company.
- Step 2 - Reconciliation:
  - The translated version is reviewed by a panel consisting ofbilingual native speakers and a researcher, to ensure full understanding and appropriate selection of words.
- Step 3 – Back Translation:
  - The reconciled version of the translated questionnaire is then back translated into English by a speaker whose mother tongue is English and who is blinded to the original version (professional translation service).
- Step 4 – Back Translation Review:
  - The English back translated version is then compared to the original questionnaire. Those items where a discrepancy between the original and back translated version have been found are then revised. Pre-final version is then ready for final review.
- Step 5 – Final review and proof reading:
  - A small group of individuals representative of the target population complete the pre-final version of the measure. Further modifications are carried out as necessary and the final version is proofread for spelling, grammatical, diacritical, or other errors.

For other research documents given to participants (such as, consent form, participant information sheet, advert leaflets), a simplified procedure of forward translation (by a professional company) followed by a review by a bilingual panel of individuals representative of the target population will be employed.

#### Incentives for participants and SFSC programmes

As a gesture of thanks, participants will receive a £10 gift voucher at the baseline interview, followed by £10 upon completion of the post-intervention questionnaire and £20 at completion of the final six-month questionnaire. Participants involved in any process evaluation interviews will receive an additional £10 voucher. SFSC programmes involved in the pilot phase of the study and delivered by community organisations received a £250 voucher as a gesture of thanks. In addition, excess treatment costs will be available to participating services to support costs associated with delivering programmes involved in the main trial.

#### Participant withdrawal

Participants have the right to withdraw from the study at any point without needing to give a reason or explanation. This will not affect participants’ ability to access and complete the SFSC programme, as explained in the participant information sheet.

### Intervention

SFSC is a group based universal parenting programme designed to support parents with children aged up to 18 years to improve their well-being, confidence and competence in parenting; develop better relationships with their children; explore strategies to put appropriate boundaries in place; support their children to minimise risky behaviours; and help children transition through childhood to adulthood. SFSC aims to help parents gain a better understanding of their child’s development and help promote self-esteem and social skills in their children. It also aims to empower parents to play a more active role in their local communities.

Based upon social learning theory, it uses interactive methods to encourage parents to share their experiences and undertake practical activities during the sessions as well as at home, to develop their skills, confidence and self-esteem. SFSC is structured into five component areas which are incorporated throughout the 13-week programme: cultural/ spiritual; rite of passage; enhancing relationships; process of discipline; and community involvement.

SFSC is delivered by two trained practitioners guided by a detailed programme manual to ensure implementation fidelity, and is supported by an extensive quality assurance process. SFSC has received the CAN parent Quality Mark [26]. All facilitators receive a 5-day training course provided by the Race Equality Foundation and gain accreditation based on a self-assessment portfolio and/ or programme observations. All facilitators who are part of the research study are expected to attend a two-day facilitator refresher training.

The programme runs for 13 weeks with each session lasting 3 h. The majority of programmes are delivered during term time only, meaning that programmes start in September; January or April. A group size between 8 and 12 parents is recommended. Parents receive a parent manual to help them understand how to implement the different concepts and this is available in multiple languages.

#### Control group

The control arm of this trial will comprise a waiting list control where the participants randomised to the control arm will be offered the programme after approximately 10 months (after the final follow-up data collection). All study participants in both trial arms will continue to have access to a full range of locally available health and social care services including Child and Adolescent Mental Health Services (CAMHS).

### Outcome measures

**Fig. 2:**
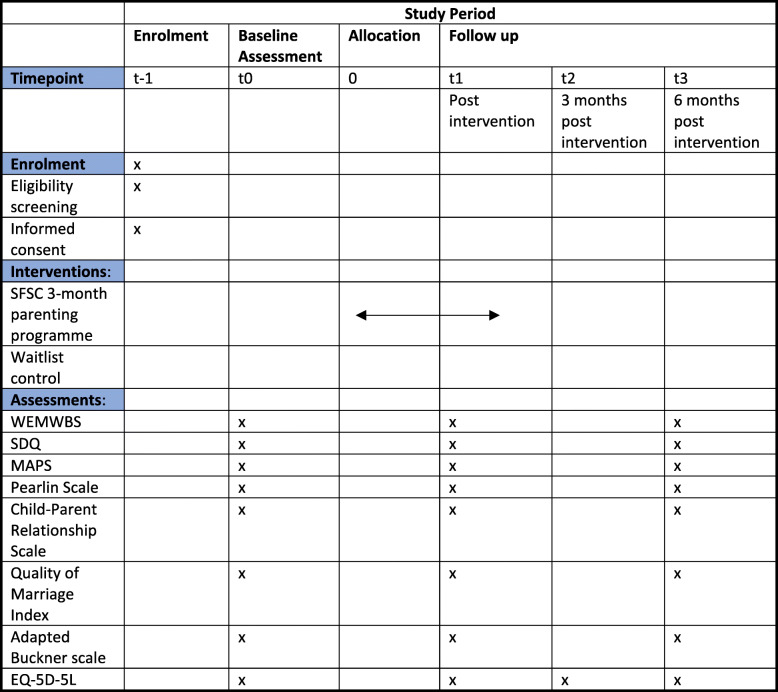
Time schedule of enrolment, interventions, and assessments for participants. t, timepoint; t − 1, enrolment of participants; T0, baseline assessment; 0 allocation to study group; t − 1, post intervention assessment; t2, 3 month follow up assessment; t3, 6 month follow up assessment

Outcome data will be collected at baseline (time point 0), post intervention (time point 1), 3 months post intervention (time point 2) and then 6 months post intervention (time point 3) (see Fig. 2). A range of validated and reliable measures have been selected to measure parent and child outcomes. The primary outcome measure, parental mental well-being, is assessed using the Warwick-Edinburgh Mental Well-Being Scale (WEMWBS) [27]**,** a 14 item self-report measure of positive mental health and wellbeing. The main secondary outcome measure, child socio-emotional well-being, is measured using the Strengths and Difficulties Questionnaire (SDQ) – parent report [28]. Other secondary outcomes include: parenting practices, measured by the Multidimensional Assessment of Parenting Scale (MAPS) [29]; self-efficacy, measured by the Pearlin Scale [30]; child-parent relationship, measured by the Child-Parent Relationship Scale [31]; family relationships and conflict, measured by the Quality of Marriage Index [32]; community cohesion, measured by the adapted Buckner scale [33]; and health-related quality of life, measured by the EQ-5D-5L [34].

### Sample size calculation

We calculated that a sample of 676 participants was needed to detect an effect size of 0.3 with 90% power at a 5% significance level and assuming an intraclass correlation coefficient (ICC) of 0.05 [35], including 20% attrition rate. This assumed 26 clusters of 13 participants per cluster within the intervention arm (*n* = 338) and 338 participants recruited to the waiting list control arm (1:1 allocation ratio). An effect size of 0.3 on the WEMWBS is indicative of a change of 3 points on the scale stated as clinically relevant by the scale manual [36] and assuming a standard deviation of 10 as seen in a similar population [37]. For the total SDQ score, the key secondary outcome, a minimal clinically significant change is 2 as indicated by Ford and colleagues [38]. Taking a SD of 6 from normative data held by ‘*youthinmind’* [39], then an effect size of 0.33 is indicated. If the ICC is assumed equivalent to that of the parental outcome, then a sample of 676 indicates 87% power; with a reduced ICC of 0.025, this power increases to 92%.

### Statistical and economic analysis

Analysis will be conducted on an intention to treat basis. The main analysis will be mixed effects models adjusted for baseline score, allocation group, and stratification variables. Site will be included as a random effect. To minimise bias due to missing data, predictors of missingness will be investigated using regression models and any predictors found will be considered for inclusion in the models. Multiple imputation will be employed to address missing scores where appropriate. Analysis of complete case data will be carried out as a sensitivity analysis to establish the sensitivity of the treatment effect estimates to the missing data. All treatment effect estimates will be presented with 95% confidence intervals. Additional regression analyses will explore factors associated with effectiveness. Exploratory analysis will initially look at including a measure of adherence within the models to assess the levels of effectiveness. Depending on the exact socio-demographic nature and diversity of the sample once the recruitment stage is completed, exploratory sub-group analysis will investigate different aspects of inequalities in the effectiveness of the intervention across the study population. Information with regard to data entry, coding, security, and storage is documented in a data management plan, which is available on request.

An economic evaluation will be conducted, following the recommendations of the NICE Public Health Reference Case [40]. The analysis will take a public sector perspective, estimating cost and cost effectiveness for the within-trial period. Cost effectiveness will be evaluated using a cost-utility analysis (CUA) and cost-consequences analysis (CCA). We will undertake a detailed cost analysis to estimate the costs of delivering the SFSC programme, including: development and training of accredited providers; the cost of delivering the group sessions; participant monitoring activities; and any follow-up/management. Broader resource utilisation over and above the costs of the SFSC programme will be captured via parent questionnaires administered at each data collection time point. Resource use data will be collected on NHS, social care, criminal justice system, and education contacts. Unit costs will be derived from local and national sources and estimated in line with best practice. Costs will be standardised to constant prices.

Outcomes for the economic evaluation will include WEMWBS (the primary outcome in the trial), the full range of secondary outcomes, and parent quality-adjusted life years (QALYs); all of these will be included in the CCA; the last of these will be used to undertake a CUA. For this, parent health-related quality of life, measured at baseline, end of programme, and 3 and 6 months post intervention using the EuroQol EQ-5D-5L will be converted into health utilities using established utility algorithms to estimate QALYs [41]. Parent-specific utility profiles will be constructed assuming a straight-line relation between each of the participant’s EQ-5D scores at each follow-up point. The QALYs experienced from baseline to final follow-up will be calculated as the area underneath this profile. We will calculate QALYs for all parents involved in the study; we acknowledge that some families in the study will be single-parent families, but the proportion of single-parent families will be similar in both trial arms. We will not include child QALYs given that some children in the trial may be as young as 3 years of age and there are no validated health-related quality of life measures in young children.

Cost effectiveness in the CUA will be calculated as the mean cost difference between the SFSC programme versus control divided by the mean difference in QALYs to give the incremental cost effectiveness ratio (ICER). We will undertake extensive sensitivity analyses. Non-parametric bootstrap estimation will be used to derive 95% confidence intervals for mean cost differences between the trial groups and to calculate 95% confidence intervals for incremental cost effectiveness ratios [42]. The bootstrap replications will also be used to construct a cost-effectiveness acceptability curve, which will show the probability the SFSC programme is cost-effective for different values of the public sector’s willingness to pay for an additional QALY. A series of deterministic sensitivity analyses will explore the implications of uncertainty on the incremental cost effectiveness ratios.

### Process evaluation

In line with MRC guidelines [43, 44] an embedded mixed methods process evaluation will be implemented to assess intervention fidelity and provide a contextualised analysis of intervention delivery.

During the first stage of the study, the process evaluation focused on the feasibility and acceptability of the study procedures. Quantitative programme activity data was collected to assess adherence to fidelity and recruitment targets, while qualitative interviews with participants (*n* = 8) and staff (*n* = 12) were conducted to investigate the acceptability of study procedures and the recruitment and randomisation processes. We gave particular attention to attitudes towards randomisation to the waiting list control group.

During the main trial**,** the process evaluation will seek to describe the implementation of the intervention, continue to assess intervention fidelity and implement a multi-perspective qualitative investigation of intervention delivery. Routine data is collected on programme recruitment, registration, referrals, attendance, retention and staffing. Data is also collected on participation, reach, dose received (number of sessions attended), and retention rate (overall rates and in particular with reference to socio-economic position and ethnic diversity of sample). Fidelity measures are completed by staff delivering the programme at the end of each session. Additionally, an adapted version of the Parent Programme Implementation Checklist [45] will be used to assess fidelity during observation visits carried out by the research team in a random sample of programmes.

During the main trial, qualitative data collection will be extended to include individual semi-structured interviews with lead commissioners (*n* = 7) and SFSC programme coordinators (*n* = 7) across each area. In addition, we will complete individual semi-structured interviews with staff and participants, including staff involved in delivering the programme across the seven areas (*n* = 21) and participants (*n* = 20). The participants will be a purposive sample selected to represent range and diversity in terms of age of index child, ethnicity and other demographic characteristics.

#### Analysis of process evaluation data

Quantitative data on fidelity and engagement (attendance and retention) and service activity will be subject to descriptive analyses. Qualitative interviews will be audio recorded, professionally verbatim transcribed and translated where necessary before being subjected to a thematic analysis [46]. This will be supported by use of Nvivo and involve initial data organisation through coding, category development and then testing. Analysis will focus in particular on exploring the range of views on the relevance, appropriateness and acceptability of the programme, perceived barriers and facilitators to change in parenting approaches, how the programme helped or hindered this in the family environment, and examine whether, and how, intervention delivery and outcome generation are influenced, either positively or negatively, by contextual factors. In this way the process evaluation will generate empirical data which will enable mechanisms of action to be described and the underlying theory of change to be evaluated. The work will be undertaken in parallel with the trial and will support interpretation of trial outcome data, thereby increasing the explanatory potential of the study while also potentially informing strategies for downstream implementation of the intervention.

### Patient and public involvement (PPI)

Public involvement is essential in all stages of public health research. It is particularly important in the evaluation of complex interventions that seek to support and empower families living in challenging circumstances, and from marginalised communities to further develop their parenting skills and abilities. The study includes four main strands to PPI:

1. Development stage - A PPI co-applicant (KM) has been involved in the initial development of the study, bringing ‘expert by experience’ as a parent and previous participant in the SFSC programme. KM is also a member of the Trial Management Group.
2. Trial Steering Committee - Two lay members of the public are members of the Trial Steering Committee providing advice and support on the conduct of the trial from a lay and parental perspective.
3. Parent advisory groups (PAGs) – three groups comprising of 8–12 previous participants of the SFSC programme have been established in three of the study locations (South London, North London and Manchester). The groups meet three times a year during the study period, facilitated by experienced members of the study team. The members of the advisory groups are reflective of the parents who usually attend SFSC and our target study population group (i.e. parents live in deprived and/or ethnically diverse areas). In recognising that parents may lack confidence in undertaking this role, each parent is individually supported and encouraged to perform their role, and any support needs identified before meetings (i.e. translation needs). Each group also had an introductory training session designed to demonstrate how their input can ensure that the research is relevant, practical and to build their confidence in expressing their views. The PAGs provide invaluable input into all aspects of the study but in particular provide insights on the best ways to engage with potential participants, recruitment strategies, formatting and design of outcome measures and reporting and dissemination of study results. PAG activities are documented and regularly evaluated to assess their impact on the study.
4. Adolescent and young people’s forum – One group of 8 adolescents and young people aged 14–18 years whose parent(s) previously attended a SFSC programme has been established. The adolescent and young people’s forums specifically explore their views and perspectives of the SFSC programme and its perceived impact on their parent(s), family dynamics and their own behaviours and feelings.

### Ethics

Ethical approval for the study was given by the UCL Research Ethics Committee (reference 1538/002) on the 27th of February 2019.

### Trial supervision and oversight

Two independent oversight committees (Trial Steering Committee and Data Monitoring and Ethics Committee) have been established to oversee the supervision and governance of the trial. In addition, a Trial Management Group oversees the on-going implementation and conduct of the study. These committees and group function in accordance with the NWORTH, Bangor CTU standard operating procedures.

#### Initial implementation of the trial: insights and challenges

In the next section, we report on the implementation of the trial in the internal pilot and the challenges and adaptations to the original protocol that have occurred to date.

#### Progression to full trial

RAG (red/amber/green) progression criteria [47] were used to assess the randomisation, retention and intervention attendance to determine whether the stage 2 main trial could proceed (see Table 1). In line with the RAG criteria outline, ACCEPT criteria were used to determine whether pilot data could be included in the main trial [48].

**Table 1:** RAG progression criteria

Indicator	Green	Amber	Red
Randomisation: 130 participants, either: (a) across all 5 sites/programmes OR (b) if one or two sites significantly underperforms, across the best four or three sites	More than 80%	40–80%	Less than 40%
Retention: at 3-months post intervention (around 6-months from baseline)	More than 85%	50–85%	Less than 50%
Attendance: proportion attending at least nine out of the 13 programme sessions	More than 70%	50–70%	Less than 50%
Completeness of data: on an outcome measure at baseline and post-intervention	Less than 20% missing data		
Intervention fidelity	Completion of facilitator self-assessment and quality assessment		

Based on the progression RAG criteria, progression to full trial was warranted. The total number of randomisations across the five internal pilot sites for the first 6 months of recruitment was 123, which is 95% of the target for this period and achieves a progression criterion of green. Only 14 participants withdrew from the internal pilot study prior to the 3 months follow-up. Therefore, the retention rate for the 3 months follow-up was 89% (109/123), which achieves a progression criterion of green. The median number of sessions of the SFSC programme attended by participants was 9 out of 13. The percentage of participants attending nine or more sessions was 51%, which achieves a progression criterion of amber.

Data completion - For both timepoints, and for all outcome measures the percentage of missing data was substantially less than 20%. Hence, all outcome measures are deemed acceptable for the full-scale trial.

Intervention fidelity - Overall, the feedback from facilitators on the SFSC programme regarding the quality and fidelity of the intervention was positive. Participants were motivated and engaged. Participants bonded well with other group members and were willing to share their real-life experiences with the group. Notably, the sessions with the community speaker were typically well received. However, for some sessions there were issues with low attendance, sessions over-running and participants being distracted.

#### Interviews with participants and delivery staff

This section draws on analysis from qualitative interviews with eight parents who took part in the internal pilot and 12 members of staff involved in commissioning or delivering SFSC during the pilot phase.

Parents appeared to accept and understand the randomisation process although the reasons for the study design were not always fully understood. Parents were generally accepting of the waitlist design. This view was echoed by the research team who found that most parents they recruited accepted the chance they may be allocated to the waitlist. Services on the other hand expressed some reservations about the randomisation process. Services were sometimes found to “gatekeep” participants by deciding which parents to invite for the study based on their assessment of need. Ongoing communications between the research team and members of staff was vital and the research team learned that it is of upmost importance that services understand, and are committed to being part of the research (and the randomisation process) before taking part. For engagement with new sites the research team will ensure that services only take part when fully comfortable with the randomisation process. To increase the understanding and acceptance of the randomisation process, as well as to maintain enthusiasm and engagement with the research, the research team has introduced quarterly study newsletters and delivered webinars. During these webinars, we discuss the research and encourage services to learn from each other’s experiences and hear from PPI members about their views on the research processes.

Staff interviews and reflections from the research team also revealed that it is vital that those delivering the programme are fully aware of their role in the research. Facilitators stated that their managers had not always informed them of their part in the research study from the start, creating some resistance. The research team will ensure that in communications with commissioners, service managers and decision makers it is stressed that facilitators should be involved early on in discussions and made aware of their part in the research. Similarly, some facilitators work on a freelance basis and although refresher training and research trainings are offered for free, facilitators were slightly reluctant to offer their time to engage with training without being compensated. The research team will use the excess treatment costs to compensate freelance facilitators for their part in the main trial.

#### Reflections from research team

As is common in most trials, recruitment of parents into the trial was challenging at times. Although services are asked to recruit twice as many parents into their programme as usual, initial discussions with Local Authorities and community organisations delivering SFSC suggested they felt confident in recruiting the required numbers within a relatively short period (4 weeks). The research team’s experience to date is that services overestimate the number of parents on their waitlist and that a wider and more structured outreach plan to recruit parents is necessary. We have worked hard to improve the recruitment strategy during the early stages of the trial. The interviews with parents and feedback from PPI members suggest that we should aim to appeal to people’s altruism when asking them to be a part of the study. We have therefore updated the recruitment materials accordingly. We have widened the recruitment window and ask services to start outreach earlier than they normally would. We now promote different approaches within services such as recruitment events, the use of local schools, social media, online events and engagement with referrers.

#### Impact of the COVID-19 pandemic

The emergence of the COVID-19 pandemic created challenges for the trial. In line with government social distancing and lockdown guidelines, all face-to face-delivery of the SFSC programme was paused between March 2020 and September 2020. Face-to-face delivery and recruitment into the trial resumed in November 2020 but the intervention delivery, method of recruitment and data collection have been modified to minimise transmission of the virus and protect participants, research staff and service delivery staff. Ethics approval was granted to make the following changes which are included in the latest protocol version 4 (16th February, 2021):

- Intervention delivery – SFSC programmes that are running face to face can offer online group support to those participants who are not able to attend due to COVID-19 restrictions.
- Recruitment – baseline interviews are conducted via video or telephone calls. Consent is obtained remotely by asking participants to return a digital copy of the consent form and, if this is not possible, through audio recording the consent process by reading through the consent form with the participant, and asking them to respond to each point. These recordings are subsequently stored securely in encrypted files.
- Data collection – all data are collected remotely via video call or telephone call when video call is not possible. Ethical approval was gained for the change in recruitment and data collection methods.
- Sample size - Due to the impact of COVID-19 social distancing restrictions, the sample size has been reviewed and revised to 672 participants (360 intervention: 312 control). The changes undertaken still accommodate the power to assess the effects originally planned. The sample initially allowed for clustering within both arms, despite there being none in the control arm, the inflation in the control arm was kept to accommodate an anticipated differential drop out. This differential drop out was not evident in the internal pilot and therefore this inflation has been removed. The recalculation also allows for variation in the size of the recruited groups from 6 to 13 to accommodate the varying group sizes due to social distancing restrictions on venues. Recalculation of the sample therefore results in an unequal allocation ratio with the optimal allocation ratio of 1.154 to 1 being employed.

## Discussion

The TOGETHER trial aims to provide evidence of the effectiveness and cost effectiveness of the SFSC parenting intervention for parents of children aged between 3 and 18 years. The trial is rigorously designed with a waitlist control and blinded outcome assessment.

Supporting parents to develop effective parenting practices is an important core strategy to tackle inequalities in childhood and adolescence but major gaps remain in the evidence base for universal parenting programmes, especially for older children and families from disadvantaged and diverse ethnic backgrounds. The results of this trial evaluating the SFSC programme will therefore provide useful data to inform future action to address ethnic and socio-economic inequalities in child health and well-being.

To the best of our knowledge, this is the first time that the SFSC programme has been evaluated using a RCT design. Although RCTs remain the ‘gold standard’ to examine the effectiveness of an intervention, a key strength of the study is the nested mixed methods process evaluation to provide a comprehensive contextual evaluation of the intervention to understand what, how, and for whom SFSC may be beneficial. Another strength of the study is the inclusion of SFSC programmes delivered by native non-English speakers trained as SFSC facilitators, often highly popular in the minority communities. While parents accessing these programmes would often be excluded from research and therefore under-represented in research evidence due to language and cultural barriers, being inclusive was paramount in the present study and made possible by translating all relevant research documents and training native speakers from local communities to collect data.

As described in earlier sections, recruitment into the trial is slower than originally anticipated. Although the COVID-19 pandemic has presented significant challenges to the delivery of SFSC and group sizes, the fact that the programme only runs during school terms and therefore only three times a year presents challenges for recruitment. During school holidays it is particularly hard to reach parents. Nevertheless, with a combination of remote and face-to-face recruitment methods this may become easier for the remainder of trial.

## Conclusion

Randomised controlled trials of complex interventions are challenging to run, particularly in the current context of the COVID-19 pandemic. Nonetheless, this study is on course to succeed due to a highly committed research team, a strong partnership with the Race Equality Foundation, dedicated services who appreciate the added value of this research, and impressive engagement with supportive PPI members. This trial fits in with the NIHR’s priority of community-based trials that aim to be inclusive and reduce health inequalities. Furthermore, by conducting this study, we hope to add to the literature of low-intensity parenting programmes and build the evidence base for SFSC.

## Data Availability

At the end of the study, NWORTH CTU will release a data pack containing the raw data extracted from MACRO database, the analysis data sets and any agreed syntax. The Statistical Analysis Plan, data and code can be shared publicly upon reasonable justified request.

## Abbreviations

CCA: Cost consequences analysis
CUA: Cost utility analysis
DMEC: Data monitoring and ethics committee
EAP: Economic analysis plan
ICC: Intra cluster correlation
MCS: Millennium Cohort Study
MRC: Medical Research Council
NIHR: National Institute for Health Research
PPI: Patient and public involvement
QALYs: Quality adjusted life years
RCT: Randomised controlled trial
REF: Race Equality Foundation
SAP: Statistical analysis plan
SDQ: Strengths and Difficulties Questionnaire
SFSC: Strengthening families, strengthening communities
TMG: Trial management group
TSC: Trial steering committee
WEMWBS: Warwick and Edinburgh Mental Well Being Scale
